# An analysis of timing and frequency of malaria infection during pregnancy in relation to the risk of low birth weight, anaemia and perinatal mortality in Burkina Faso

**DOI:** 10.1186/1475-2875-11-71

**Published:** 2012-03-16

**Authors:** Innocent Valea, Halidou Tinto, Maxime K Drabo, Lieven Huybregts, Hermann Sorgho, Jean-Bosco Ouedraogo, Robert T Guiguemde, Jean Pierre van Geertruyden, Patrick Kolsteren, Umberto D'Alessandro

**Affiliations:** 1 Laboratory of Parasitology and Entomology, Centre Muraz, Bobo-Dioulasso, Burkina Faso; 2 Institut de Recherche en Sciences de la Santé, Direction Régionale de l'Ouest, Bobo-Dioulasso, Burkina Faso; 3 Department of Food Safety and Food Quality, Ghent University, Ghent, Belgium; 4 Department of International Health, University of Antwerp, Antwerp, Belgium; 5 Unit of Nutrition and Child Health, Department of Public Health, Prince Leopold Institute of Tropical Medicine, Antwerp, Belgium; 6 Unit of Epidemiology and Control of Parasitic Diseases, Department of Parasitology, Prince Leopold Institute of Tropical Medicine, Antwerp, Belgium

**Keywords:** Malaria infection, Pregnancy, First trimester, Sulphadoxine-pyrimethamine, IPT

## Abstract

**Background:**

A prospective study aiming at assessing the effect of adding a third dose sulphadoxine-pyrimethamine (SP) to the standard two-dose intermittent preventive treatment for pregnant women was carried out in Hounde, Burkina Faso, between March 2006 and July 2008. Pregnant women were identified as earlier as possible during pregnancy through a network of home visitors, referred to the health facilities for inclusion and followed up until delivery.

**Methods:**

Study participants were enrolled at antenatal care (ANC) visits and randomized to receive either two or three doses of SP at the appropriate time. Women were visited daily and a blood slide was collected when there was fever (body temperature > 37.5°C) or history of fever. Women were encouraged to attend ANC and deliver in the health centre, where the new-born was examined and weighed. The timing and frequency of malaria infection was analysed in relation to the risk of low birth weight, maternal anaemia and perinatal mortality.

**Results:**

Data on birth weight and haemoglobin were available for 1,034 women. The incidence of malaria infections was significantly lower in women having received three instead of two doses of SP. Occurrence of first malaria infection during the first or second trimester was associated with a higher risk of low birth weight: incidence rate ratios of 3.56 (*p *< 0.001) and 1.72 (*p *= 0.034), respectively. After adjusting for possible confounding factors, the risk remained significantly higher for the infection in the first trimester of pregnancy (adjusted incidence rate ratio = 2.07, *p *= 0.002). The risk of maternal anaemia and perinatal mortality was not associated with the timing of first malaria infection.

**Conclusion:**

Malaria infection during first trimester of pregnancy is associated to a higher risk of low birth weight. Women should be encouraged to use long-lasting insecticidal nets before and throughout their pregnancy.

## Background

Each year, about 30 million pregnant women are at risk for malaria [1], with consequences of public health concern [2]. Indeed, malaria infection may result in maternal anaemia [3], pre-term delivery and low birth weight (LBW) [2,4] and is therefore an important determinant of perinatal mortality [5-7]. To prevent and manage malaria in pregnancy, the Word Health Organization (WHO) recommends effective case management, use of insecticide-treated bed nets and intermittent preventive treatment (IPTp) with sulphadoxine-pyrimethamine (SP) [8]. Following the latest WHO guidelines, IPTp should be administered at least twice, during the second and third trimester. In Burkina Faso in 2005, the Ministry of Health (MoH) replaced weekly chloroquine chemoprophylaxis, whose efficacy had declined because of widespread resistance [9], with IPTp/SP [10]. Several studies have demonstrated the excellent safety and efficacy of IPTp [11-15]. Most studies used maternal anaemia and LBW as impact indicators. IPTp prevents the adverse effects of malaria during pregnancy by clearing any active infection and providing post-treatment prophylaxis. The length of protection may become shorter and infection may occur progressively earlier as SP resistance increases [16]. As SP is given at least twice during pregnancy, its prophylactic efficacy may vary according to both the timing of malaria infection and SP administration. Unfortunately, because of fears of possible toxicity, SP cannot be given during the first trimester of pregnancy. However, there is also little information on the consequence of malaria infection during the first trimester as most information available refers to infections occurring in the second and third trimester [17-20]. A randomized trial comparing two doses *versus *three doses of IPTp/SP was carried out in rural Burkina Faso. Participants were recruited as early as possible during pregnancy and followed-up prospectively until delivery, allowing an estimation of the effect of malaria infection at different gestational ages on the risk of maternal anaemia, LBW and perinatal mortality. Results are reported below.

## Methods

### Study settings

Two peripheral health centres (Koho and Karaba) in the Houndé health district, south-west Burkina-Faso, were selected for the study. In this area, malaria is markedly seasonal with high transmission during the rainy season (June to December) [21]. The district hospital and the 28 peripheral health facilities cover a population of approximately 247,500 people. In 2007, the number of pregnant women at risk of malaria was estimated at 12,500, while malaria was the main disease in the health district, accounting for about 38% of all consultations and 52% of hospitalizations.

### Study design

This was part of a larger study investigating both the effect of multiple micronutrients supplementation (MMS) *versus *fortified food supplementation (FFS) and that of IPTp/SP, two *versus *three doses on the health of pregnant women and that of their offspring. Participants were randomized in permuted blocks of four to receive either two doses of SP as recommended by the National Malaria Control Programme (NMCP) or three doses. Randomization numbers were generated by a computer program, sealed in opaque envelopes and opened only when an eligible subject was identified. These numbers were then transmitted to the study pharmacist who packaged the drugs in individual plastic zip bags. Each bag was labelled with the participant's name, identification, residence, and randomization group. The field pharmacist prepared for each woman an individual schedule for SP administration according to the gestational age at randomization and transmitted it to the trained home visitors who administered both SP and either MMS or FSS. Results of the nutrition intervention have been reported elsewhere [22]. The effect of IPTp with three doses of SP *versus *two doses of SP on LBW and pregnancy outcomes have also been reported in a previous publication [23].

### Study participants

Pregnant women were identified through a community-based network of home visitors, as described elsewhere [22]. All women of child-bearing age in the study area were identified and paid monthly visits during which they were screened for pregnancy. Women suspected to be pregnant were referred to the health facilities for a pregnancy test. The study protocol and procedures were then explained to the potential participants in the local language and those agreeing to participate were asked to provide a written inform consent. Women with known hypersensibility to SP or not planning to stay in the study area for the next two years were excluded.

### Recruitment and follow-up

Study participants were recruited at ANC clinics, where the health staff recorded demographic data, medical and pregnancy history. In addition, a clinical examination was performed and vital signs, weight, height and arm circumference were measured. All measures were repeated at each ANC visit. Gestational age was assessed as early as possible by the study obstetrician using trans-abdominal ultrasound fetal biometry. Women included in the study were visited at home daily by the home visitors who recorded the body temperature and registered any complaint. In case of fever (body temperature ≥ 37.5°C) or history of fever since the last visit, a blood sample for thick and thin film was collected and sent to the health district laboratory. All women with a confirmed malaria infection were treated with a full course of quinine (24 mg/kg/day for seven days), regardless of their gestational age. Pregnant women were encouraged to attend their scheduled ANC visits and to deliver at the health facilities where the new-borns was examined, weighed and measured twice by two different members of the health staff.

### Laboratory methods

Thick and thin blood smears were collected in duplicate and stained with Giemsa 10% (pH 7.2) for 10 minutes. Parasite densities were determined on the thick smears by counting asexual parasites per 200 white blood cells (WBC) and assuming a WBC count of 8,000/μl. A thick blood smear was considered negative after reading 100 high-power fields. Ten percent of the slides were randomly selected and sent to the Laboratory of Parasitology/Centre Muraz for quality control. Haemoglobin (Hb) was measured by using a portable spectrophotometer (HemoCue, Angelholm, Sweden).

### Ethics

The study protocol was approved by the ethical committees of the Centre Muraz, Bobo-Dioulasso, Burkina Faso, and the University of Antwerp, Belgium. The trial was registered in the ClinicalTrial.gov registry (identifier: NCT00909974). The study purpose and procedures where explained to the potential participants by the study clinician in the local languages. Women who fulfilled the inclusion criteria and agreed to participate in the study were asked to provide a written inform consent.

### Definitions and statistical analysis

A new-born was classified as LBW if the birth weight was < 2,500 g. Women with haemoglobin levels < 11 g/dl were classified as anaemic, those with haemoglobin levels < 8 g/dl were classified as having moderate-to-severe anaemia. Neonatal death was defined as one occurring between delivery and 28 days of life. Malaria infection was defined as a slide positive for *Plasmodium falciparum*, any density. Incidence rates were computed considering the follow-up duration time (in months) of each woman. Women with a malaria infection after previous treatment with quinine were considered as re-infected. Time to re-infection was defined as the period between the first infection parasite clearance and the second infection. As the parasite clearance was not monitored, we calculated an adjusted time to re-infection considering that all parasites were cleared after three days as indicated in a previous publication [24].

Data were double entered in a Microsoft Access^® ^database by two data clerks. Validation and analysis were performed using Stata 10 IC^® ^software. Only singleton pregnancies were included in the analysis. The *Chi2 *or Fisher exact test were used to compare proportions for categorical variables while a t-test for normally distributed or Mann-Whitney test for non-normally distributed was used for continuous variables at baseline. A multivariate analysis using a Poisson regression model with robust standard error estimates to evaluate the relationship between explanatory variables and outcomes was also performed. Crude and adjusted incidence rate ratios (IRR) with 95% CI were computed. A *p*-value ≤ 0.05 was considered as statistically significant.

The timing of malaria infection was defined according to the first infection detected at the first, second or third trimester of pregnancy. The frequency of malaria infection was categorized according to the number of positive slides detected during the whole pregnancy, i.e. 0, 1, or ≥ 2. Explanatory variables and possible confounders included the number of SP doses received (two or three), the nutritional supplementation (FFS or MMS), gravidity, body mass index at enrolment (underweight: body mass index < 18.5 kg/m^2^; or normal weigh, BMI ≥ 18.5 kg/m^2^), and malaria transmission season at delivery (low transmission season between December and May and high transmission season between June and November).

## Results

### Study population

From March 2006 to July 2008, 1,296 women were randomized into the study. Table 1 shows the baseline characteristics of study population according to the timing of first infection. Characteristics of study participants according to randomization groups were published elsewhere [23]. A total of 1,053 women attended at least three ANC visits while 121 and 100 attended two and one ANC visit, respectively. Analysis was performed on 1,034 women with complete records on birth weight and haemoglobin. The mean maternal age was 24.4 years and the mean gestational age at enrolment was 15.9 weeks. There was no difference in terms of baselines characteristics between women included in the analysis and those excluded for missing data.

**Table 1:** Characteristic of study participants at inclusion by timing of first infection

Characteristics	No infection (n = 816)	Infection in first trimester (n = 31)	Infection in second trimester (n = 69)	Infection in third trimester (n = 118)
Study location				
Karaba village (n, %)	219 (26.84)	14 (45.16)	20(33.90)	40(33.94)
Koho village (n, %)	597 (73.16)	17 (54,84)	49(66.10)	78(66.06)

Education				
none (n, %)	728 (89.21)	22 (70.97)	62(89.86)	105 (88.99)
primary (n, %)	69 (8.46)	8 (25.81)	7(10.14)	10 (8.47)
secondary and higher (n, %)	19(2.33)	1 (3.22)	0(0)	3(2.54)

Parity				
primigravidae (n, %)	113 (13.85)	18 (58.06)	35 (50.72)	44 (37.29)
secundigravidae (n, %)	158 (19.36)	5 (16.13)	15 (21.74)	38 (32.20)
multigravidae (n, %)	545 (66.79)	8 (25.81)	19 (27.54)	36 (30.51)

Age				
mean (years) (95% CI)	25.27(24.84-25.69)	20.88 (18.96-22.80)	21.27 (20.06-22.48)	21.50 (20.63-22.37)

Weight				
mean (kg) (95% CI]	55.67 (55.17-56.15)	55.45 (53.20-57.71)	55.24 (53.70-56.79)	55.29 (54.08-56.50)

Height	162.815	163.39	162.58	161.78
mean (cm) (95% CI]	(162.42-163.21)	(161.72-165.08)	(161.15-164.00)	(160.71-162.85)

BMI at enrolment	20.98	20.77	20.88	21.1
mean (95% CI]	(20.83-21.12)	(19.97-21.56)	(20.41-21.34)	(20.74-21.45)

### Incidence rates of malaria detected infections

The overall incidence rate (IR) of malaria infection (expressed per 1,000 women-months) was 39.2, and was significantly higher in primi- (88.6, 95%CI: 72.6-108.1) than in secundi- (50.6, 95%CI: 39.1-65.5) and multi-gravidae (18.8, 95%CI: 14.7-24.1) (Table 2). The IR of malaria infection was significantly lower (*p *= 0.014) in women having received three (23.8%, 95%CI: 15.5-36.4) than two SP doses (46.6%, 95%CI: 38.3-55.4). No difference between the MMS group (40.4, 95%CI: 33.5-48.6) and the FFS group (38.0%, 95%CI: 31.5-45.9) was found (*p *= 0.42).

**Table 2:** Number of cases and incidence rates of malaria infections in the study population (per 1000 women-month)

	Person-time*		Malaria infections	
		
		new cases	IR (per 1000) (95% CI)	IRR (95% CI)
By Gravidity				
Overall	5541	217	39.2 (34.3-44.7)	-
Primigravidae	1095	97	88.6 (72.6-108.1)	-
Secondigravidae	1146	58	50.6 (39.1-65.56)	0.6 (0.42-0.79)
Multigravidae	3300	62	18.8 (14.65-24.1)	0.2 (0.17-0.31)

By SP doses received				
2 doses SP	2453	113	46.1 (38.3-55.4)	-
3 doses SP	884	21	23.8 (15.5-36.4)	0.5 (0.28-0.66)

By Supplementation group				
MMS	2726	110	40.3 (33.5-48.6)	-
FSS	2815	107	38.0 (31.5-46.0)	0.9 (0.67-1.18)

* Women-month

### Rates of re-infection and length of time-to-reinfection

The overall incidence of re-infections after quinine treatment was 11.4% (95%CI: 8.9-14.6) and was significantly higher (*p *< 0.001) in primi- (34.7, 95%CI: 25.3-47.7) than in secundi- (11.3, 95%CI: 6.6-19.5) and multi-gravidae (3.6%, 95%CI: 2.1-6.4). Re-infection tended to be lower in women having received three (6.8%, 95%CI: 3.1-15.1) than two (14.3%, 95%CI: 10.2-19.9) SP doses (*p *= 0.06). No difference between MMS and FFS group was found (data not shown).

Overall, the adjusted mean time to re-infection was 59.3 days (95%CI: 45.8-72.7) with no difference by gravidity, i.e. primi- 63.7 days (95%CI: 47.2-80.2), secundi- 40.7 days (95%CI: 07.9-73.4), and multi-gravidae 51.2 days (95%CI: 3.2-99.1). Mean time to reinfection was not analysed by number of SP doses as only four women in the three-dose group were re-infected.

### Effect of timing and frequency of malaria infection on low birth weight, maternal anaemia and perinatal mortality

When considering the timing of the first infection, no significant difference could be found for maternal anemia and perinatal mortality, while women infected both in the first (IRR = 3.56, *p *< 0.001) and the second trimester (IRR = 1.72, *p *= 0.034) had a significantly higher risk of delivering a LBW baby as compared to women without any infection (Table 3). After adjusting for age, parity, body mass index, number of SP doses received and malaria season at delivery, such risk remained significantly higher for women infected in the first trimester of pregnancy (adjusted IRR = 2.07, *p *= 0.002) (Table 3). Also, women infected in first trimester had a significantly higher risk of delivering a LBW when compared to women infected in the third trimester (Crude IRR = 3.09, 95%CI: 1.66-5.73, *p *< 0.01; and adjusted IRR = 3.27, 95%CI: 1.78-6.01, *p *< 0.01) while no difference was observed between women infected in the second trimester and women infected in the third trimester (Crude IRR = 1.49, 95%CI: 0.78-2.87, p = 0.23 and Adjusted IRR = 1.58, 95%CI: 0.83-2.98; p = 0.16).

**Table 3:** Crude and adjusted incidence rate ratios (IRR) of low birth weight, maternal anaemia and perinatal mortality by trimester of malaria infection using Poisson regression

	Crude IRR (95%CI)	Adjusted IRR(95%CI)
**Maternal anaemia**		
No infection (391/816)	1.00	1.00
Infection in 1^st ^trimester (15/31)	1.00 (0.69-1.46)	0.98 (0.67-1.42)
Infection in 2^nd ^trimester (34/69)	1.03 (0.80-1.32)	0.99 (0.76-1.27)
Infection in 3^rd ^trimester (58/118)	1.02 (0.84-1.24)	0.97 (0.79-1.20)

**Low birth weight**		
No infection(96/816)	1.00	1.00
Infection in 1^st ^trimester (13/31)	3.56 (2.26-5.61) ^**a**^	2.07 (1.30-3.27) ^**b**^
Infection in 2^nd ^trimester (14/69)	1.72 (1.04-2.85) ^**c**^	1.09 (0.65-1.82)
Infection in 3^rd ^trimester (16/118)	1.15 (0.70-1.88)	0.78 (0.46-1.32)

**Perinatal mortality**		
No infection(15/816)	1.00	1.00
Infection in 1^st ^trimester (0/31)	-	-
Infection in 2^nd ^trimester (3/69)	2.36 (0.70-7.97)	1.18 (0.31-4.47)
Infection in 3^rd ^trimester (1/118)	0.46 (0.06-3.46)	0.37 (0.04-2.81)

Adjusted IRR accounted for women age, parity, BMI, SP doses received and delivery season. ^a^*p *< 0.001, ^b ^*p *= 0.002, ^c ^*p *= 0.034

Most women had just one infection though some of them experienced up to four infections until delivery. Though the LBW risk seemed to be significantly higher in women with two or more infections as shown by the crude IRR, the difference disappeared after adjusting for women age, parity, body mass index, number of SP doses received and malaria season at delivery (Table 4). Similarly, the risk for maternal anaemia, though apparently increased by two or more infection, did not vary significantly with the number of re-infections after adjustment by the variables mentioned above. The risk of perinatal mortality was similar for women with ≥ 1 infection and those with no infection (adjusted IRR = 0.87, 95% CI 0.27-2.83) (Table 4).

**Table 4:** Crude and adjusted incident rate ratios (IRR) of low birth weight, maternal anaemia and perinatal mortality by number of detected malaria infection using Poisson regression

	Crude IRR (95%CI)	Adjusted IRR(95%CI)
**Maternal anaemia**		
No infection (391/816)	1.00	1.00
1 infection detected (79/169)	1.07 (0.91-1.26)	1.019 (0.85-1.21)
≥ 2 infections detected (28/49)	1.33 (1.04-1.69)^**a**^	1.236 (0.94-1.62)

**Low birth weight**		
No infection (96/816)	1.00	1.00
1 infection detected (31/169)	1.56 (1.08-2.26)^**b**^	1.07 (0.73-1.58)
≥ 2 infections detected (12/49)	2.08 (1.29-3.52)^**c**^	1.08 (0.62-1.87)

**Perinatal mortality**		
No infection (15/816)	1.00	1.00
1 infection detected (4/169)	1.28 (0.43-3.83)	0.87 (0.27-2.83)
≥ 2 infections detected (0/49)	-	-

Adjusted IRR accounted for women age, parity, BMI, SP doses received and delivery season. ^a ^*p *= 0.023, ^b ^*p *= 0.19, ^c ^*p *= 0.006

## Discussion

The risk of delivering a LBW baby was significantly higher in women infected during the first trimester of pregnancy, even after adjusting for several potential confounding variables such as parity and number of IPTp/SP doses received. Thought the pathogenesis and immunity of malaria in pregnancy has been explored, the contribution of placental immuno-pathology to anaemia and LBW is not fully understood [25]. *Plasmodium falciparum*-infected erythrocytes sequester in the placenta through adhesion mechanisms, inducing placental inflammatory responses, particularly monocytes infiltrates. Inflammatory cytokines produced by T cell and macrophages, in particular Th1 responses are associated with maternal anaemia, spontaneous abortions and premature deliveries. These cytokines are known to help eliminate the parasites from the placenta but their overproduction can threaten the pregnancy [25,26]. Acute infection, particularly with high parasites densities have been associated with preterm delivery while chronic infection have been associated with LBW due to intrauterine growth retardation (IUGR) and severe anaemia [27]. Decreased placental growth and/or decreased nutrient transport have been suggested as the possible final common pathways by which malaria leads to IUGR [25,28]. In this study, malaria infection during the first trimester was strongly associated with LBW. It is unclear whether this is due to a specific consequence of the infection at this particular time, or whether it is related to the higher risk these women have throughout their pregnancy. In any case, the currently recommended IPT/SP does not cover the first trimester of pregnancy as the first SP dose should not be given before quickening, at about 20 weeks of gestation, for fear of possible toxicity for the foetus [29]. There is little information on the use of SP during the first trimester of pregnancy; available reports are limited to small case series from developed countries [15]. Therefore, the only option available for protecting pregnant women during this vulnerable period is the use of long-lasting insecticidal nets (LLIN) whose coverage and use in sub-Saharan Africa, despite major efforts such as campaigns of free mass distribution, is less than optimal [30,31]. A potentially interesting additional strategy might be the systematic screening and treatment of all pregnant women and this option was recently investigated [32,33]. However, when considering the difficulty of identifying women at the earliest stage of their pregnancy [34], such an approach would hardly solve the problem of protecting during the first trimester. Therefore, promoting LLIN through community-based promotional campaigns targeted not only at pregnant women but also at adolescents seems to be the only option available to tackle this specific problem.

The rate of re-infection and the time length to re-infection were suggested as possible indicators to evaluate the preventive efficacy of SP [35]. In this study, the incidence of re-infections was much higher in women having received two as compared to three doses of SP, indicating that the latter is probably more efficacious. Nevertheless, the rate of re-infection was established after treatment with quinine and does not necessarily represent the true preventive efficacy of SP.

The study has some limitations. Information on LLIN ownership and use was not collected and, knowing that this has an impact on the malaria risk during pregnancy [36], it should probably have been considered as an important confounding factor. However, it is unlikely that this has resulted in biased estimates as LLIN use in the study population was probably very low at the time of the study. In Boromo, a neighbouring district, LLIN use was estimated at about 27% in the high transmission season [21].

Malaria infection during pregnancy was probably underestimated as it was detected by peripheral blood smears. Placental biopsies were collected during the study but could not be analyzed due to budget constraints.

Despite the network of home visitors implemented and the daily visits carried out for an earlier identification of pregnant women, most of those detected during the first trimester were at ≥ 10 weeks of gestation, a limiting factor for the assessment of infections during the first trimester. For some reasons, mainly socio-cultural factors [34], women were reluctant to declare their pregnancy earlier and this should be further investigated.

In conclusion, malaria infections occurring in the first trimester of pregnancy seem to have an important effect on LBW, though the mechanisms are not yet understood. Women should be encouraged to use LLIN throughout their pregnancy so that the deleterious effect of malaria infection during the first trimester could be prevented.

## Competing interests

The authors declare that they have no competing interests.

## Authors' contributions

IV participated in study coordination and data cleaning, performed the data analysis and drafted the manuscript. HT participated in the design, study coordination and corrected the manuscript. MKD, HS and JPG corrected the manuscript. LH participated in data cleaning and corrected the manuscript. RTG and KP participated in the design of the study. UDA participated in the design, helped in data analysis and corrected the manuscript. All authors read and approved the final manuscript.
